# Societal perceptions of caregivers linked to culture across 20 countries: Evidence from a 10-billion-word database

**DOI:** 10.1371/journal.pone.0251161

**Published:** 2021-07-01

**Authors:** Reuben Ng, Nicole Indran

**Affiliations:** 1 Lee Kuan Yew School of Public Policy, National University of Singapore, Singapore, Singapore; 2 Lloyd’s Register Foundation Institute for the Public Understanding of Risk, National University of Singapore, Singapore, Singapore; University of West London, UNITED KINGDOM

## Abstract

Caregivers play an indispensable role in society. In 2017, 41 million family caregivers in the United States provided approximately 34 billion hours of care to their aging parents, spouses, partners and friends. The estimated economic value of their unpaid contributions amounted to $470 billion. Despite their invaluable contributions, caregivers often operate in a reality of inadequate social support. Little is known about the factors linked to the societal perceptions of caregivers, and our study seeks to contribute by filling this gap. Importantly, whether society honors or stigmatizes caregivers is critical as it could impact caregiving decisions and either exacerbate or ameliorate caregiver burden. We leveraged an online media database of 10 billion words collated from over 28 million articles, across 20 countries, to analyze societal perceptions of caregivers. Of the 20 countries, 18 evidenced neutral to positive perceptions of caregivers. Bangladesh and Ghana had the most positive perceptions, while Sri Lanka and Tanzania had the most negative perceptions. Aging demographics and culture (individualism, masculinity and uncertainty avoidance) were significantly associated with perceptions of caregivers. Findings suggest that positive perceptions of caregivers can be better cultivated when caring is lauded as a virtuous act that aids in reducing the care deficit. This study is among the first to analyze the societal perceptions of caregivers globally, and lays the groundwork to design culturally sensitive interventions that increase society’s appreciation for caregivers.

## Introduction

Caregivers play an indispensable role in society [1]. In 2017, 41 million family caregivers in the United States provided approximately 34 billion hours of care to their aging parents, spouses, partners and friends. The estimated economic value of their unpaid contributions amounted to $470 billion [2]. Despite their invaluable contributions, there are few studies on societal perceptions of caregivers. Whether society honors or stigmatizes caregivers is critical as it could impact caregiving decisions [3] and either exacerbate or ameliorate caregiver burden. Our study fills this gap by analyzing societal perceptions of caregivers across 20 countries in an online media database of 10 billon words collated from over 28 million articles. In light of the global nature of the dataset, we hypothesize that societal perceptions of caregivers will be linked to country-level demographic variables (prevalence of aging and speed of population aging) and cultural factors (Hofstede’s widely used dimensions: individualism, masculinity, power distance and uncertainty avoidance).

This study is significant in three ways. Firstly, from a conceptual standpoint, we contribute to the field of cultural gerontology by investigating the cultural and demographic factors that shape societal perceptions of caregivers. Through this, we provide insight into the societal standing of caregivers—how societies view caregivers across the demographic continuum. Secondly, we uncover the cultural underpinnings of stereotypes associated with caregivers—an area of literature that remains under-explored. The expansive nature of our 20-country study allows us to distill the critical aspects of culture that determine whether caregivers are honored or stigmatized in various countries. Thirdly and practically, the role of caregivers will become even more crucial since the worldwide demand for long-term care will continue to increase [4]. Societal perceptions of caregivers will have profound implications on the caregiving experience, to the extent of either mitigating or exacerbating the strains of caregiving. Support from society—both formal and informal—will help to alleviate caregiver burden [5] and promote successful caregiving [6]. Positive societal perceptions offer a conducive environment for caregiving practices to be sustained, while improving the quality of life of both caregivers and care recipients [7]. We synthesize the literature that provided the theoretical basis for our hypotheses.

### Cultural and demographic influences of perceptions towards caregivers

The majority of studies on caregivers focus on self-perceptions and burden, with few on societal perceptions of caregivers. The effects of caregiving are commonly discussed in relation to caregiver burden. Notwithstanding the benefits of caregiving such as personal fulfilment and a sense of purpose, caregiving frequently exacts a huge toll on caregivers’ psychological well-being [8]. Besides having to deal with the often distressing experience of caregiving, they also perform the difficult balancing act of fulfilling work, social, household and caregiving commitments, especially in the case of young adults who make up an increasingly large subset of caregivers [9]. Caregivers may also grapple with income loss or limited career prospects as they cut back on work to carry out their caregiving duties [6, 9]. On top of this, they generally face greater risk of social isolation, which tends to eventuate in reduced social support over time [10]. In addition, depression among caregivers of patients with cancer [11], disabilities [12] and severe mental illnesses [13] has become increasingly prevalent over time.

Societal perceptions may impact self-perceptions and caregiving decisions. For example, a caregiver’s psychological burden may worsen if caregiving is perceived negatively or undervalued. The value ascribed to caregiving may be influenced by the way in which care is culturally constructed in society. Previous studies have examined the role of cultural values and traditions in informing motivations to provide care [14–17]. In East Asia, where cultural norms regarding filial piety regulate family behavior, looking after one’s aging parents is a matter of honor and duty [18]. Allowing professionals to take over this responsibility might be frowned upon as an act of negligence [18]. Studies indicated that the strong culture of caring in Japan can be credited to the high value assigned to care—a core value of the country shaped largely by the concept of *omoiyari* (empathy). However, another study found that caregivers in Japan are motivated more strongly by the desire to avoid negative social sanction in the form of shame [19]. This fear of shame is rooted in the concept of *sekentei*, which refers to social appearance, reputation or dignity in the community, and serves as a principal factor in the decision to provide care. Beyond the East Asian context, the concept of *familismo* (familism) in Latin America emphasizes the importance of caring for one’s family, prioritizing the needs of family members, as well as respecting older family members [20]. Hence, for Latin Americans, caregiving is an embodiment of these virtues, making it an emblem of cultural pride [20]. Similarly, African Americans’ high involvement in caregiving can be attributed to sociocultural beliefs regarding reciprocity within the family [15]. It has also been suggested that norms of solidarity in various cultures undergird caregiving decisions, particularly in the context of intergenerational relationships [17]. We describe our study’s cultural framework.

Hofstede’s [21] model of cultural dimensions was first developed in a study conducted among IBM employees in 72 countries. Despite criticisms, subsequent studies continue to support the validity of his findings [22]. We find his framework meaningful for the following reasons. Firstly, the multidimensional framework offers extensive coverage of cultural variations as opposed to any single dimension. Secondly, it has been widely utilized, ensuring this study’s comparability with and contribution to earlier work. Thirdly, Hofstede’s national-level (between-system) measurement of culture is appropriate for our country-level analysis [23].

Although Hofstede’s model has since been refined, his original four-dimensional framework proves most relevant to our study. The four dimensions are power distance, individualism–collectivism, masculinity–femininity and uncertainty avoidance. Power distance is defined as “the extent to which the less powerful members of organizations and institutions accept and expect that power is distributed unequally. The basic problem involved is the degree of human inequality that underlies the functioning of each particular society.” Individualism–collectivism refers to “the degree to which individuals are supposed to look after themselves or remain integrated into groups, usually around the family. Positioning itself between these poles is a very basic problem all societies face.” Masculinity–femininity is “the distribution of emotional roles between the genders, which is another fundamental problem for any society to which a range of solutions are found; it opposes “tough” masculine to “tender” feminine societies.” Uncertainty avoidance indicates “the extent to which a culture programs its members to feel either uncomfortable or comfortable in unstructured situations. Unstructured situations are novel, unknown, surprising, different from usual. The basic problem involved is the degree to which a society tries to control the uncontrollable.”

At present, there are no known studies at the national level concerning the interplay of individualism, masculinity, uncertainty avoidance and perceptions of caregivers. However, possible links can be found in existing literature. Cultures with strong individualistic tendencies have been said to emphasize personal achievement and self-sufficiency [24]. Such cultures define the self as an independent, autonomous and distinct unit. Self-reliance is considered a strength, while seeking help is thought to imply weakness [25]. In contrast, cultures that lean towards collectivism generally espouse interdependence and group solidarity [26]. There are studies on how collectivistic systems and individualistic systems differ vastly in terms of expectations to provide care for the family [27]. Specifically, individualists are more likely to view caring for one’s parents as burdensome and prefer to rely on formal services. Meanwhile, caregiving in collectivistic societies is anchored in sociocultural beliefs surrounding familial obligations [27]. Research on the relationship between individualism–collectivism and perceptions of caregivers is scant. Nevertheless, scholars have alluded to the individualistic aversion to caregivers [24, 28, 29]. Specifically, freedom in individualistic societies involves giving and receiving as little care as possible [29]. Not only do these societies reject the notion of interdependence, they may also fear that the short-term need for assistance will lead to chronic dependence [28]. In such a situation, care recipients are deemed morally defective [28] while those who engage in caring practices are seen as undesirable [24]. Conversely, caregiving is an unquestioned norm in collectivistic cultures that prioritize familial responsibility [16]. We hypothesize that countries with higher individualism scores will be associated with negative sentiments towards caregivers (Hypothesis 1).

The majority of research pertaining to the masculinity–femininity dimension is situated in the context of advertising, business, decision-making and organizational behavior [30]. Masculine societies prize ambition and competition, while feminine societies are oriented towards cooperation, modesty and quality of life [23]. Thus far, no explicit link has been made between the degree of masculinity of a country and perceptions of caregivers. However, it is well documented in the literature that caregiving is a responsibility that tends to fall on the shoulders of women [6], and such work often goes unacknowledged [1]. Given how women’s care work is systemically undervalued, we hypothesize that countries typified by a high degree of masculinity will be associated with negative sentiments towards caregivers (Hypothesis 2).

High uncertainty avoidance usually manifests as a preference for structured situations, formal rules and established norms [23]. The bulk of literature on uncertainty avoidance is concentrated in the field of clinical psychology, with a primary focus on anxiety, depression and addiction [31]. Some scholars have also pinpointed uncertainty avoidance as a prime factor in influencing organizational behavior, particularly in terms of innovation as well as receptivity to new ideas [31]. We did not find any studies linking uncertainty avoidance to societal perceptions of caregivers. Nevertheless, we hypothesize that societies high in uncertainty avoidance will be associated with positive perceptions of caregiving (Hypothesis 3). Caregiving in these societies might be a norm. It is worth noting that many countries have initiated major reforms in long-term care policies, some of which include cutbacks in professional care [17]. The recognition of a growing care deficit in society may therefore foster greater appreciation for caregivers.

Likewise, global aging trends have heightened the demand for caregivers in the long-term care sector. Against this backdrop, we hypothesize that countries with a higher percentage of older adults who are over 65 years old will be associated with positive perceptions towards caregivers (Hypothesis 4).

## Materials and methods

### Dataset

The News on the Web dataset is one of the largest international corpora containing over 28 million articles. Funded by the National Science Foundation and the National Endowment for the Humanities, data in this corpus are collected from 7,000 newspaper and magazine websites in 20 countries [32]. These countries were chosen as the original intent of the corpus was to study English linguistics across countries where English is used widely. According to cultivation theory [33], the large representation of online media captures societal perceptions of various countries, rendering the corpus useful in studying societal perceptions of caregivers. A full year’s (2017) dataset of 1.75 billion words was used for this study. Data includes six regions: North America (America, Canada), the British Isles (Ireland, United Kingdom), Oceania (Australia, New Zealand), Asia (Bangladesh, Hong Kong, India, Malaysia, Pakistan, Philippines, Singapore, Sri Lanka), Africa (Ghana, Kenya, Nigeria, South Africa, Tanzania) and the Caribbean (Jamaica).

### Measurement of perceptions of caregivers

For the target words ‘caregiver’ and ‘caregivers’ in each country, we compiled the top 200 words (collocates) that co-occurred most frequently. We acknowledge that there exists international variation in the terms used to refer to individuals who provide informal care. However, we used ‘caregiver(s)’ for two reasons. First, ‘caregiver’ evidenced the highest prevalence globally. Second, the term ‘caregiver’ was used most commonly across the 20 countries. We were also cognizant that caregiving may refer to caring for children or persons with disabilities. However, we found that geriatric caregiving was most prevalent in the dataset—over 100 times more prevalent than caregiving for those with disabilities. Narratives related to caring for children congregated around different sets of target words such as ‘childcare’ or ‘nanny’, which were not likely to overlap with words used to refer to geriatric caregiving.

The collocates were compiled based on the following criteria: (a) Lexical Proximity: collocate present within four words before or after the respective target word; (b) Relevant context: collocate referred specifically to caregiver(s) (verified by two raters); (c) Mutual Information Score of three and above: collocate was more strongly associated with the target word than other words in the corpus for that country, highlighting semantic bonding [34]. This is a novel application of concordance analysis used to identify stereotypes and is consistent with previous studies [35]. 4000 collocates (200 collocates x 20 countries) were generated and rated on a scale from 1 (very negative) to 5 (very positive). For instance, ‘unproductive’ would be rated as very negative whereas ‘happy’ would be rated as very positive. The inter-rater reliability using Cronbach’s alpha was 0.936 (95% CI: 0.931, 0.941) for the scoring method. Societal perceptions of caregivers scores for each country were determined by calculating the mean of all collocates scores per country.

### Hofstede’s four cultural dimensions

The countries have been rated on each of the four cultural dimensions. Calculations of the scores are in Hofstede’s work [23] and based on the original IBM surveys and subsequent analyses [36]. There are two steps to determining the score of a country for each dimension. Firstly, the individual survey responses to each question are tabulated at the national level. A question requiring a 5-point Likert scale has 1 to 5 points assigned to each answer. The national mean is then obtained from these answers. A question requiring a Yes/No or multiple-choice answer will have a specific answer or set of answers such as ‘Option A or Option B’ added up to derive the national percentage. Secondly, these national-level question scores are combined according to a weighted formula to obtain a country dimension score based on 3 to 8 survey questions. The weights are for balancing the importance of each survey question and generating final scores which range from 0 to 100. We divided the scores by 100 to match other variables such that they range from 0 to 1.

### Demographic variable: Aging

To test the demographic hypothesis that societal perceptions of caregivers is related to the prevalence of aging, we included the size of the above-65 population as a proportion of the total population. In addition, we included the speed of population aging—calculated by subtracting the 2007 statistic for the size of the above-65 population from the 2017 statistic in the World Development Indicators [37]—as it will be linked to more intense fiscal pressures that pay out pensions at the expense of young taxpayers [38].

### Covariate: GDP per capita

Past studies [38–43] controlled for GDP per capita to test the possible influence of level of development or modernization on ageism. In keeping with these studies, we controlled for GDP per capital to test the influence of a country’s level of development on societal perceptions of caregivers. We used a measure of logged GDP per capita from the World Development Indicators [37].

### Analytic strategy

To test the competing hypotheses of culture and demographics, we ran OLS regressions with the cultural and demographic variables as predictors and societal perceptions of caregivers as the outcome. We ran two models to ensure the robustness of our results. Model 1 included the proportion of older people aged over 65, speed of population aging, and GDP per capita. Model 2 included both aging variables, GDP per capita and four cultural variables. We checked for heteroskedasticity through visual confirmation of the residual-versus-fitted plot of the main model as well as White’s and Breusch-Pagan/Cook-Weisberg tests. All statistical analyses were performed using SPSS 25.

## Results

### Descriptive statistics: Perceptions of caregivers and cultural dimensions

**Fig 1:**
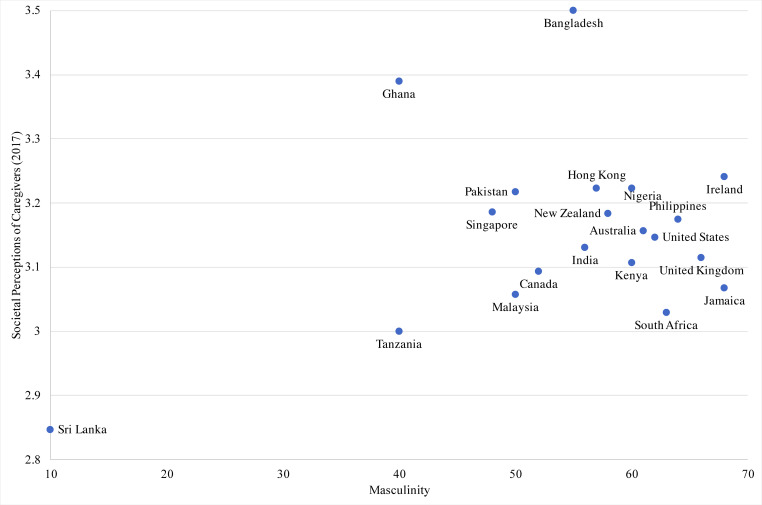
Scatterplot of societal perceptions of caregiving and masculinity across 20 countries.

**Fig 2:**
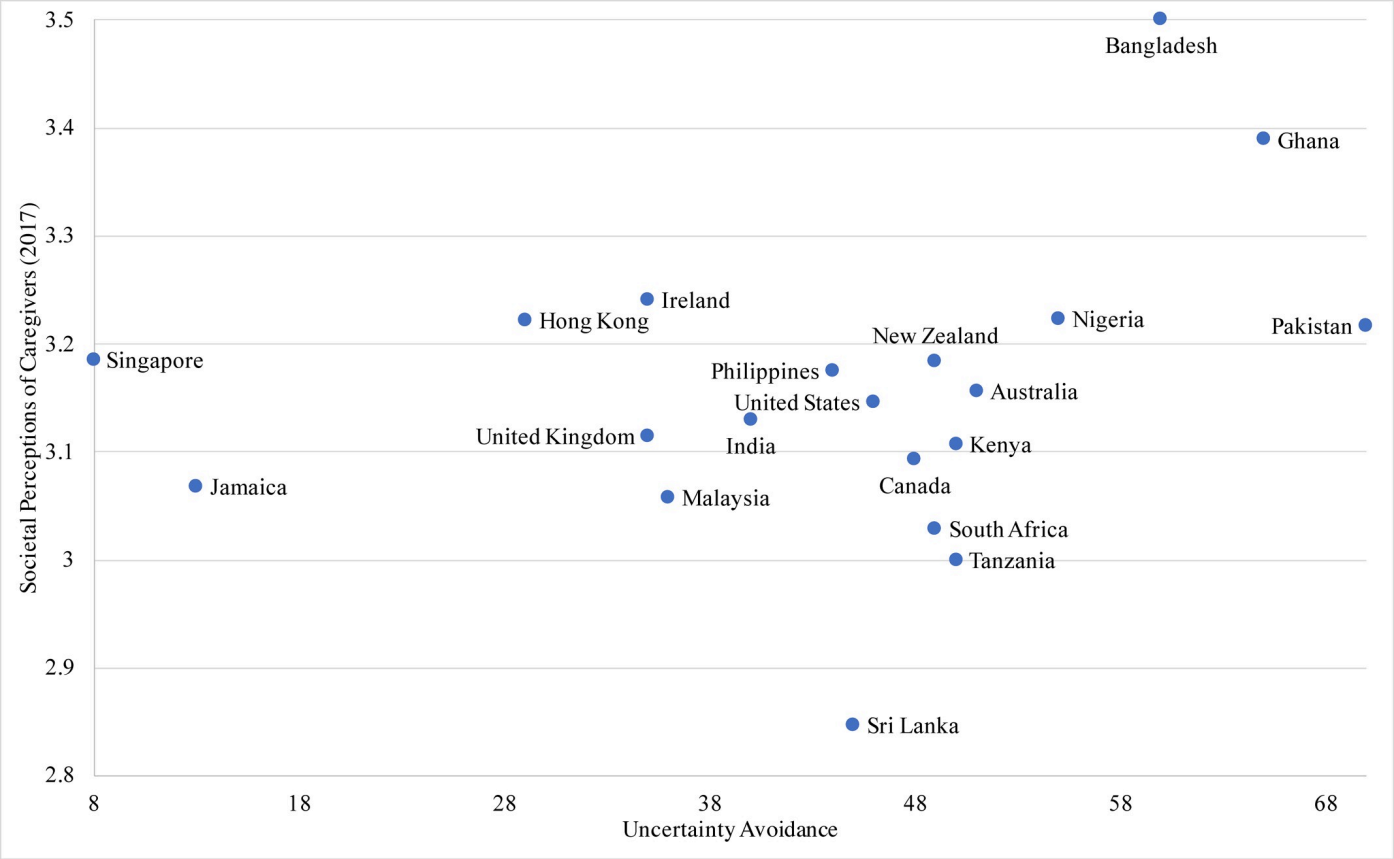
Scatterplot of societal perceptions of caregiving and uncertainty avoidance across 20 countries.

**Fig 3:**
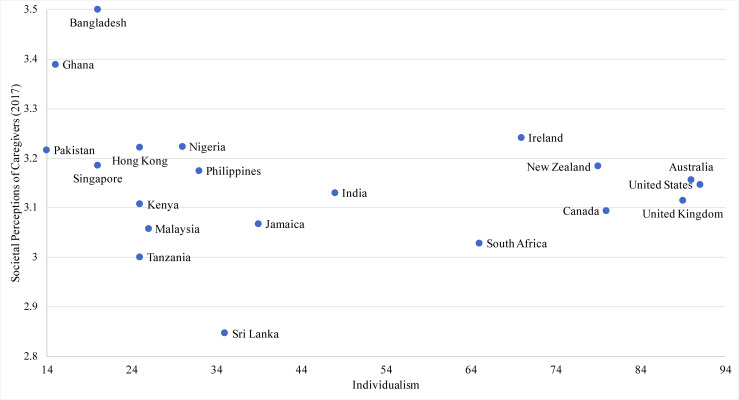
Scatterplot of societal perceptions of caregiving and individualism across 20 countries.

Of the 20 countries/territories, 18 evidenced neutral to positive views of caregivers. Sri Lanka had the most negative perceptions while Bangladesh had the most positive perceptions. The values in the sample ranged from 2.85 to 3.50. The four cultural dimensions had a theoretical range from 0 to 1, with each mean ranging from 0.44 to 0.61 and standard deviations ranging from 0.13 to 0.28. The country with the lowest power distance was New Zealand, while the highest was Malaysia. The United States scored the lowest on collectivism while Pakistan scored the highest. The country with the lowest masculinity was Sri Lanka, with Jamaica and Ireland tied as the highest. Lowest on uncertainty avoidance was Singapore and highest was Pakistan. Power distance was not significantly associated with societal perceptions of caregivers, controlling for covariates. Scatter plots for the relationships between societal perceptions of caregivers and cultural dimensions are shown as follows: Masculinity (Fig 1), Uncertainty Avoidance (Fig 2), Individualism (Fig 3).

The mean above-65 population was 9%, which is approximately the global average. The country with the largest population of older people aged over 65 was the United Kingdom (18%) while the one with the smallest was Kenya (2%). The mean change in the population aged above 65 over a 10-year period was 2%, with Hong Kong experiencing the fastest rate of aging (4%) and Nigeria displaying no aging trend (0%). This demonstrates that our sample covers a broad range of values for the demographic hypothesis.

### Multivariable regressions

We tested the hypotheses progressively across two models with Societal Perceptions of Caregiving as the outcome. Models 1 and 2 tested the demographic hypothesis that aging societies are associated with societal perceptions of caregivers. Through Model 2, our final model, we found support for hypotheses 1 through 4. Individualism was significantly associated with societal perceptions of caregiving (β = -.005, *p* = .015), likewise for Masculinity (β = .007, *p* = .008), Uncertainty Avoidance (β = .008, *p* = .005), Percentage of the population above 65 years (β = .024, *p* = .012), controlling for speed of population aging and GDP per capita (Table 1). We did not find evidence for multicollinearity as the variance inflation factors (VIF) scores were below the stringent criteria of five [44].

**Table 1:** Regressions of societal perceptions of caregivers with cultural dimensions and aging demographics.

	Models	
Variables	(1)	(2)
Population above 65	-0.002	0.024*
	(0.006)	(0.008)
Speed of population aging	9.585	3.359
	(5.191)	(4.178)
GDP per capita (log)	-0.103	-0.073
	(0.059)	(0.046)
Individualism		-0.005*
		(0.002)
Masculinity		0.007**
		(0.002)
Uncertainty avoidance		0.008**
		(0.002)
Power distance		0.001
		(0.002)
N:	20	20
R^2^:	0.187	0.687
Adjusted R^2^:	0.034	0.505

Constant not shown. Standard errors of the unstandardized coefficients are in parentheses.

** p<0.01

* p<0.05.

## Discussion

There is considerable scholarship detailing how caregivers operate in a reality of inadequate social and institutional support. However, little is known about the cultural and demographic factors that determine attitudes towards caregivers. Our study contributes by shedding light on the factors that affect how care is constructed across different cultures, and how caregivers are viewed. Through a 20-country analysis of a 10-billion-word dataset of online media, we found that Bangladesh and Ghana had the most positive perceptions of caregivers while Sri Lanka and Tanzania had the most negative perceptions. We found that both demographics (population above 65 years) and cultural dimensions (individualism, masculinity, uncertainty avoidance) were significantly associated with societal perceptions of caregivers, controlling for speed of population aging and GDP per capita.

Consistent with previous studies, albeit qualitative and conceptual, we found societies high in individualism to be associated with negative perceptions of caregivers. Individualistic societies typically champion independence and self-determination. The receipt of caregiving may represent a reliance on others, which misaligns with societal aspirations of independence [24], thus engendering negative attitudes. Furthermore, according to Gordon and colleagues [28], caregivers are “stained with shame” in societies that eschew the notion of interdependence. In such societies, the very act of caring is construed as a sign of weakness, resulting in the rejection of the legitimacy of caregivers [24].

Although no known studies have explored the link between masculinity and societal perceptions of caregivers, there are signposts that point to masculine societies being associated with more positive perceptions of caregivers. There could be two explanations. Firstly, the concept of ‘ableism’ posits that the able-bodied are glorified [41]. Societies high in masculinity may value caregivers who are more able-bodied than those they care for. Furthermore, considering the arduous nature of geriatric caregiving, caregivers may be elevated to hero status. This badge of honor may be linked to positive societal perceptions. Future studies should test ableism and heroism as mediators of the link between masculinity and perceptions of caregivers. Secondly, care work is frequently conceptualized as the responsibility of women. In Latin America for instance, women are socialized into the *marianismo* role—a role which entails deference and self-sacrifice. Performing *marianismo* involves engaging in caregiving behavior. Countries high in masculinity may therefore venerate caregivers for fulfilling gender role obligations. Additionally, some have argued that the cultural tendency to extol women’s propensity to care serves the purposes of those who benefit from women’s care. Thus, it is possible that individuals in these countries support women in their caregiving practices precisely to benefit—directly or indirectly—from women being caregivers.

Countries that are high in uncertainty avoidance are characterized by a low tolerance for ambiguity, socialized to have a high need for security and aim to be well-placed to deal with contingencies [44]. Such countries may recognize the pivotal role that caregivers play in long-term care especially as care needs become increasingly complex in aging societies. Hence, they may be more likely to normalize the role of caregivers to cope with irreversible demographic changes and rising healthcare costs, thus viewing caregivers as vital extensions of the healthcare system [8]. For instance, Ghana is high on uncertainty avoidance and has one of the most positive perceptions of caregivers. The care deficit in Ghana—brought about by societal changes such as migration, urbanization, slowed fertility and women’s increased participation in the labor market [45]—has imposed new demands on society to provide a more robust and comprehensive long-term care system within the family. Family caregivers may therefore be viewed positively for helping to plug the care deficit.

Unsurprisingly, the demographic indicator—percentage of individuals above 65 years of age—is associated with more positive societal perceptions of caregivers. The rapid growth in the population of older people has accelerated the demand for healthcare and consequently, for caregivers [46]. Caregiving in aging societies is therefore more likely to be a norm. Moreover, it is plausible that caregivers in these societies might be elevated to ‘hero status’ considering the significant role they play in long-term care [8]. This is a heartening insight that will hopefully translate to greater support for caregivers.

It is noteworthy that countries within the same geographical region (Bangladesh and Sri Lanka) and Africa (Ghana and Tanzania) scored differently on societal perceptions of caregiving. This underscores the need to consider culture, alongside geography. In Bangladesh, family caregivers perform the bulk of patient care, while nurses spend a small fraction of their duty time handling patients directly [47]. This is a result of various religious, social and cultural factors that discourage nurses—most of whom are female—from being in physical contact with ‘strangers’, particularly male patients [47]. Interestingly, caregivers look after their family members even when they are hospitalized [47]—a testament to the key role that informal caregivers play in formal healthcare, especially in acute care. Given the integration of informal and formal care provision, informal caregivers may be viewed positively.

In contrast, abuse against older adults in Sri Lanka has been identified as a pressing issue [48]. Although the country boasts a strong system of intergenerational care [49], evidence reveals it is not uncommon for family caregivers in Sri Lanka to be emotionally or verbally abusive, or to neglect their family members entirely [48]—a likely explanation for the negative attitudes towards caregivers. Multiple instances of discrimination against caregivers of mental health patients in Sri Lanka have also been reported [50]. Fernando and colleagues [50] explained that this could be partly due to ‘courtesy stigma’, where prejudice against those with mental illnesses is extended to their caregivers.

Integral to Ghanaian society is the culture of reciprocity [51, 52]. Caring for one’s parents is viewed as both a filial obligation and social responsibility. The growing care deficit in Ghana—an outcome of changing social conditions which have weakened the extended family support system—is compounded by the lack of geriatric infrastructure [45, 52]. It is thus plausible that individuals in Ghana are viewed positively for their commitment to caregiving responsibilities amid a time of rapid social change.

There is a dearth of literature on perceptions of caregivers in Tanzania and more broadly Africa—an area of research our study seeks to enrich. The negative attitudes towards caregivers in Tanzania could stem from caregivers’ association with those who are mentally ill [53], although this remains a topic ripe for future research.

Our study offers practical considerations for countries with different cultural profiles. Recently, there have been conversations on the need to operationalize the concept of kindness within the policy realm [54–56]. Kindness has been singled out as a powerful antidote to isolation and loneliness [57, 58]—both of which are public health issues that plague older adults and caregivers. Ferguson [55] suggests that we extend more care and kindness to each other such that less pressure is placed on already overburdened formal services. Given the escalating demand for caregivers, countries high in individualism could look into reframing existing notions of care. The concept of autonomy could be broadened such that the individual is viewed as a socially embedded being in an environment that elicits a fundamental need to give or receive care [59]. Rather than regarding the acceptance of care as an affront to one’s autonomy, care could be seen as a recognition of shared vulnerability [60] and an act which ultimately promotes one’s well-being [61]. The reconstruction of caregiving as a noble and rewarding undertaking could potentially encouraging more people to assume the caregiving role.

Meanwhile, it is critical that the cultural imperative to care for family members—particularly in collectivistic societies—does not breed feelings of guilt or resentment. The socially mandated nature of the caregiving role could be precisely that which allows caregivers’ efforts to go unnoticed. Attempts should therefore be made to make visible their everyday acts of service [55, 58] such that caregivers are conditioned to see their efforts as meaningful and virtuous as opposed to being merely obligatory. Social support in the form of kindness, appreciation and understanding should also be fostered at both an interpersonal and institutional level to relieve caregiver burden.

Moving forward, labor policies should normalize caregiving and encourage organizations to adopt flexible work schedules to ensure that caregivers are not sandwiched between career and caregiving. While the COVID-19 crisis has brought immense pain to various corners of society, it has also initiated work-from-home arrangements which may allow familial and professional responsibilities to be better accommodated. Flexible work schedules could increase labor force participation among caregivers while making room for existing ones to re-enter a more compassionate work environment.

Several limitations must be considered when interpreting our findings. Firstly, our database consists of only English sources and excludes super-aged societies like Japan and South Korea where caregiving is culturally ingrained. This is a significant drawback that will be overcome in future studies when the dataset is expanded. Secondly, the study was cross-sectional in design and renders any causality inference mute. Nevertheless, future studies could take a longitudinal approach to investigate the temporal predictors of societal perceptions of caregivers, and temporal stability of the cultural dimensions.

## Conclusion

Our findings suggest that positive perceptions of caregivers can be better cultivated when caring is lauded as a virtuous act that aids in reducing the care deficit. By 2030, the number of people aged 60 and above will be more than doubled. As society reaches this important demographic turning point, more people will have to take up the caregiving role. It only makes sense that caring becomes intuitive and a shared aspect of all cultures. Our study lays the groundwork for culturally sensitive interventions to be designed such that appreciation for caregivers is enhanced even as their burden is reduced.
